# Supplementary material for: Long-term effect of temporary ART initiated during primary HIV-1 infection on viral persistence
Source: Nat Commun. 2025 Jul 30;16:6989. doi: 10.1038/s41467-025-62362-0 (PMC12311192; doi:10.1038/s41467-025-62362-0)
Supplement: Supplementary file 1 — Supplementary Information [file 41467_2025_62362_MOESM1_ESM.pdf]

Supplementary Material

24-week arm

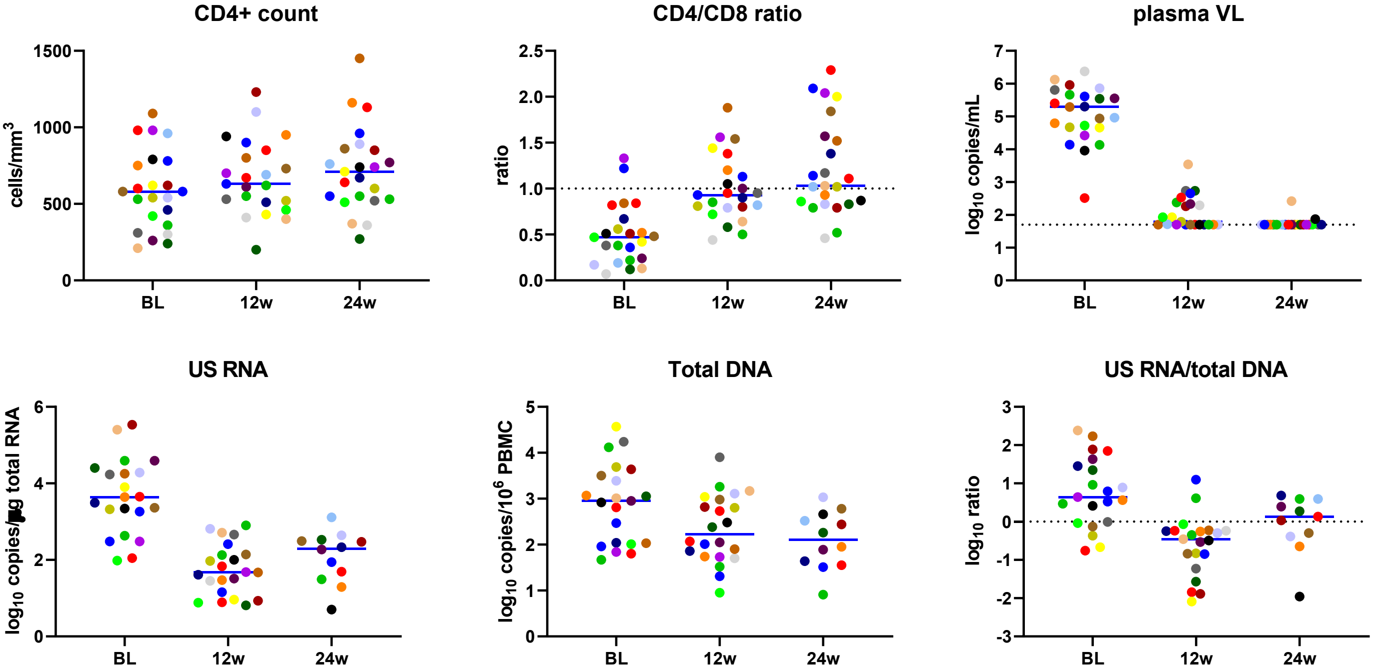

60-week arm

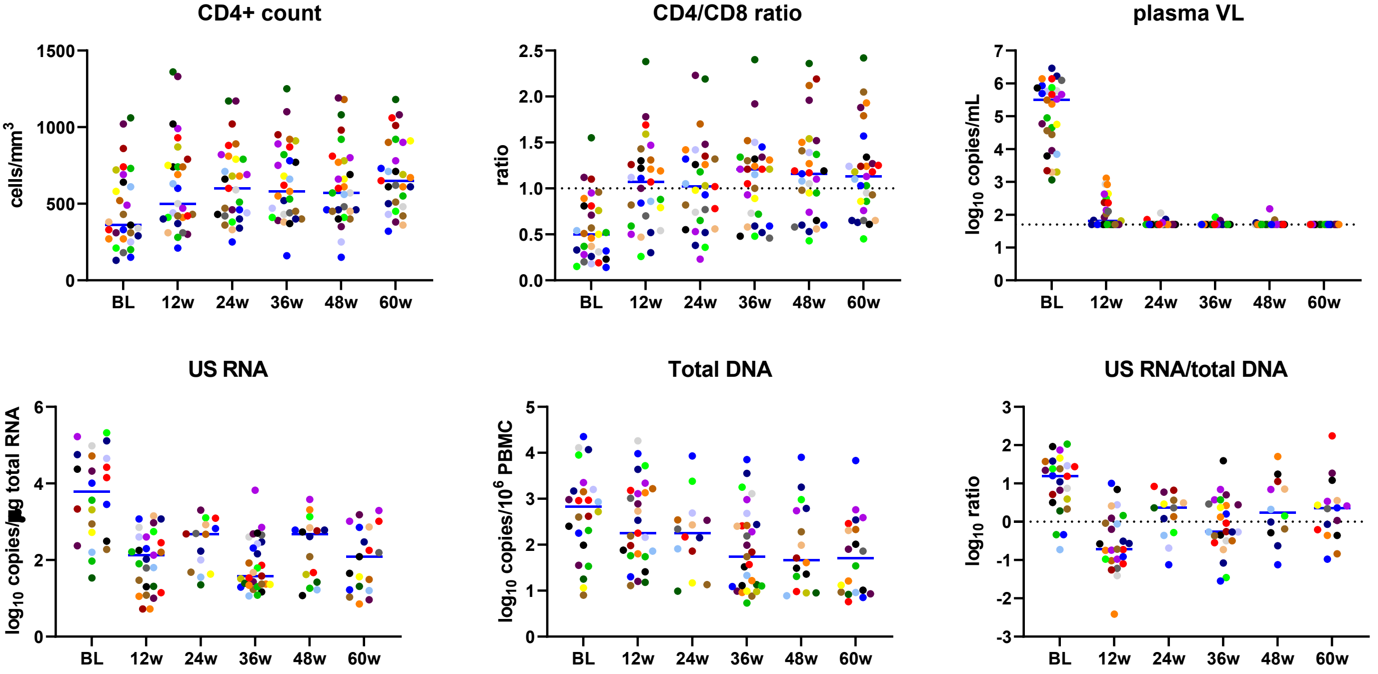

P values of 24-week arm vs. 60-week arm comparisons

	US RNA	Total DNA	US/TD ratio	Plasma VL	CD4+ count	CD4/CD8
**BL**	0.86	0.50	0.35	0.80	0.083	0.59
**12 weeks**	0.22	0.62	0.93	0.85	0.29	0.71
**24 weeks**	0.19	0.78	0.48	0.70	0.22	0.25

Figure S1

A

Early ART

CD4+ count
Total p<0.0001
BL vs. 12w p=0.0007
BL vs. 24w p<0.0001
BL vs. 36w p=0.0004
BL vs. 48w p=0.0069
BL vs. 60w p<0.0001

CD4/CD8 ratio
Total p<0.0001
BL vs. 12w p<0.0001
BL vs. 24w p<0.0001
BL vs. 36w p<0.0001
BL vs. 48w p<0.0001
BL vs. 60w p<0.0001

Plasma VL
Total p<0.0001
BL vs. 12w p<0.0001
BL vs. 24w p<0.0001
BL vs. 36w p<0.0001
BL vs. 48w p<0.0001
BL vs. 60w p<0.0001
12w vs. 24w p<0.0001
12w vs. 36w p=0.0032
12w vs. 48w p=0.0013
12w vs. 60w p=0.0005

US RNA
Total p<0.0001
BL vs. 12w p<0.0001
BL vs. 24w p<0.0001
BL vs. 36w p<0.0001
BL vs. 48w p<0.0001
BL vs. 60w p<0.0001
24w vs. 36w p=0.029
36w vs. 48w p=0.040

Total DNA
Total p=0.0029
BL vs. 12w p<0.0001
BL vs. 24w p<0.0001
BL vs. 36w p<0.0001
BL vs. 48w p=0.0002
BL vs. 60w p<0.0001
12w vs. 24w p=0.004
12w vs. 36w p<0.0001
12w vs. 48w p=0.0047
12w vs. 60w p<0.0001
24w vs. 36w p=0.026
24w vs. 60w p=0.032

US RNA/total DNA
Total p<0.0001
BL vs. 12w p<0.0001
BL vs. 24w p=0.0002
BL vs. 36w p=0.0002
12w vs. 24w p=0.014
12w vs. 60w p=0.022

B

CHI ART

CD4+ count
Total p<0.0001
BL vs. 12w p<0.0001
BL vs. 24w p<0.0001
BL vs. 36w p<0.0001
BL vs. 48w p<0.0001
BL vs. 60w p<0.0001
BL vs. 96w p<0.0001
12w vs. 36w p=0.017
12w vs. 48w p=0.0004
12w vs. 96w p<0.0001

CD4/CD8 ratio
Total p<0.0001
BL vs. 12w p<0.0001
BL vs. 24w p<0.0001
BL vs. 36w p<0.0001
BL vs. 48w p<0.0001
BL vs. 60w p<0.0001
BL vs. 96w p<0.0001
12w vs. 36w p=0.0001
12w vs. 48w p=0.001
12w vs. 60w p<0.0001
12w vs. 96w p<0.0001
24w vs. 60w p=0.012
24w vs. 96w p<0.0001
36w vs. 96w p=0.0078

Plasma VL
Total p<0.0001
BL vs. 12w p<0.0001
BL vs. 24w p<0.0001
BL vs. 36w p<0.0001
BL vs. 48w p<0.0001
BL vs. 60w p<0.0001
BL vs. 96w p<0.0001
12w vs. 24w p=0.0038
12w vs. 36w p=0.0017
12w vs. 48w p=0.037

US RNA
Total p<0.0001
BL vs. 12w p<0.0001
BL vs. 24w p<0.0001
BL vs. 36w p=0.0003
BL vs. 48w p=0.0006
BL vs. 60w p<0.0001
BL vs. 96w p<0.0001
36w vs. 60w p=0.027

Total DNA
Total p=0.020
BL vs. 12w p=0.0004
BL vs. 24w p<0.0001
BL vs. 36w p=0.0001
BL vs. 48w p=0.0015
BL vs. 60w p<0.0001
BL vs. 96w p=0.0022
12w vs. 60w p=0.016

US RNA/total DNA
Total p=0.015
BL vs. 12w p=0.0021
BL vs. 24w p=0.023
BL vs. 48w p=0.0027
BL vs. 60w p=0.011

Figure S2

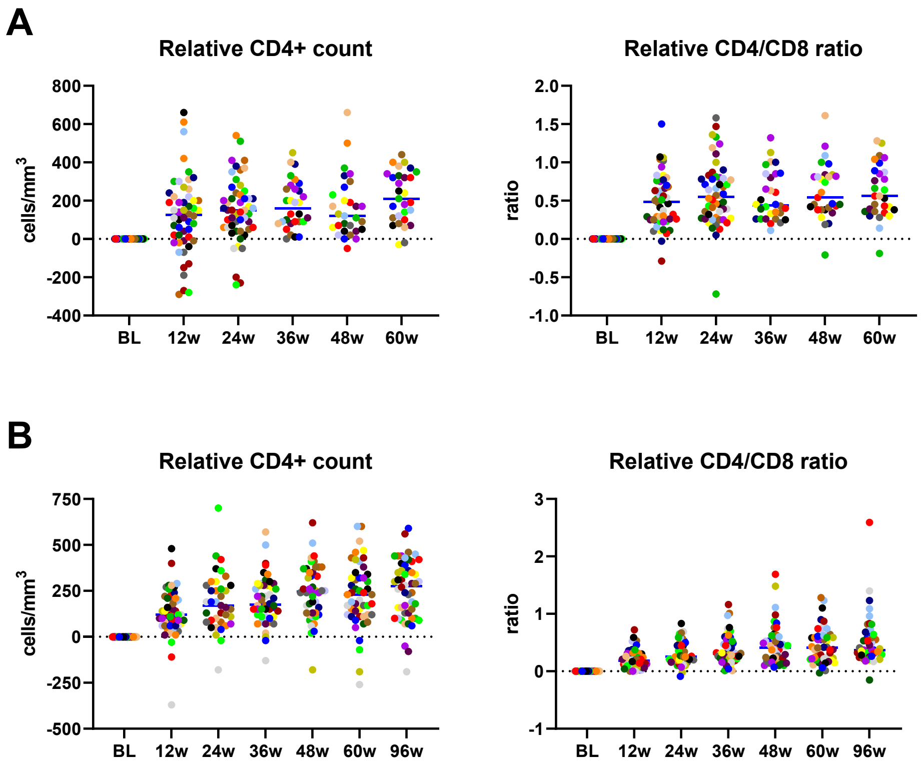
A
Relative CD4+ count
cells/mm3
800
600
400
200
0
-200
-400
BL
12w
24w
36w
48w
60w
Relative CD4/CD8 ratio
ratio
2.0
1.5
1.0
0.5
0.0
-0.5
-1.0
BL
12w
24w
36w
48w
60w
B
Relative CD4+ count
cells/mm3
750
500
250
0
-250
-500
BL
12w
24w
36w
48w
60w
96w
Relative CD4/CD8 ratio
ratio
3
2
1
0
-1
BL
12w
24w
36w
48w
60w
96w

Figure S3

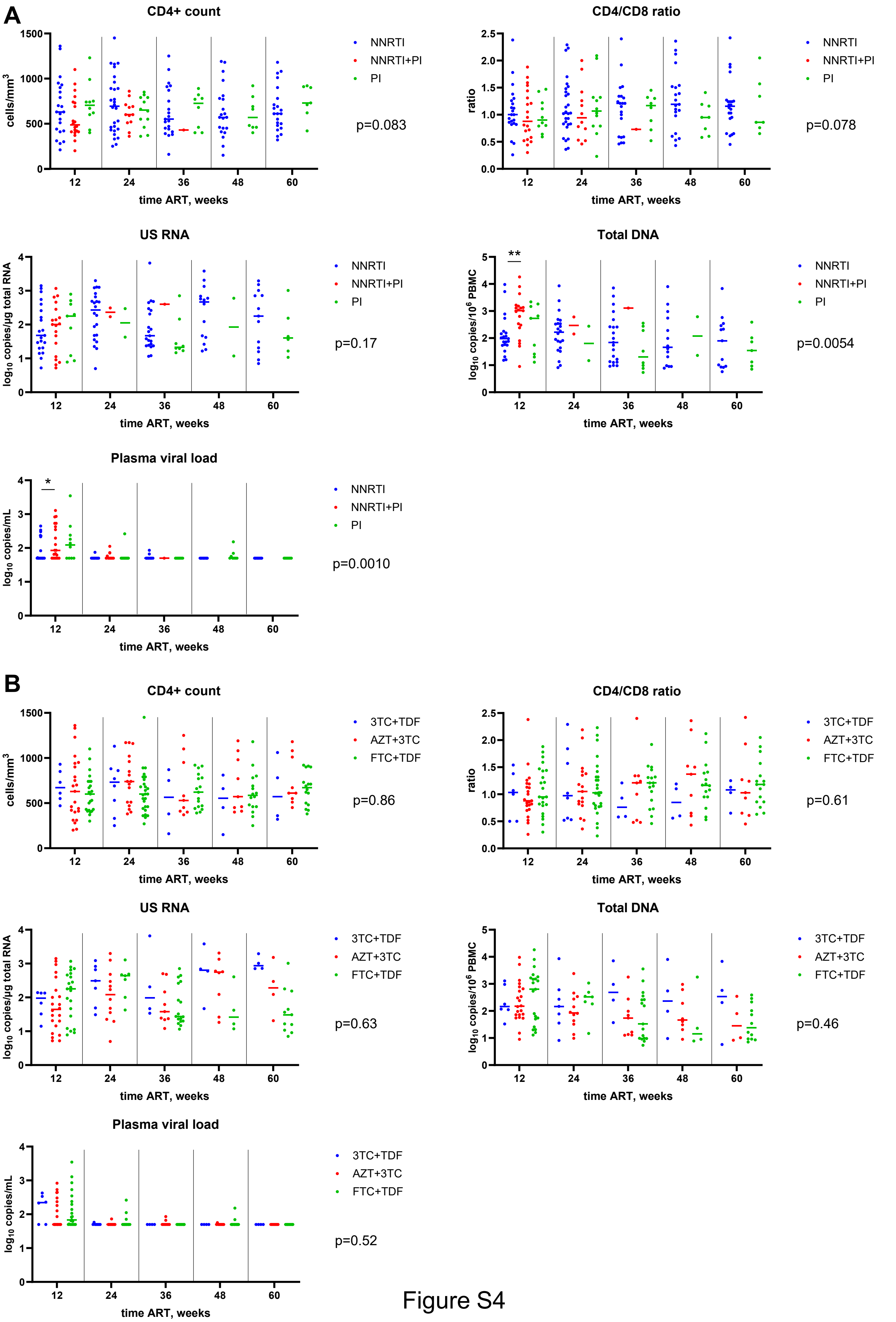
A
CD4+ count
cells/mm3
time ART, weeks
NNRTI
NNRTI+PI
PI
p=0.083
CD4/CD8 ratio
ratio
time ART, weeks
NNRTI
NNRTI+PI
PI
p=0.078
US RNA
log10 copies/µg total RNA
time ART, weeks
NNRTI
NNRTI+PI
PI
p=0.17
Total DNA
log10 copies/10^6 PBMC
**
time ART, weeks
NNRTI
NNRTI+PI
PI
p=0.0054
Plasma viral load
log10 copies/mL
*
time ART, weeks
NNRTI
NNRTI+PI
PI
p=0.0010
B
CD4+ count
cells/mm3
time ART, weeks
3TC+TDF
AZT+3TC
FTC+TDF
p=0.86
CD4/CD8 ratio
ratio
time ART, weeks
3TC+TDF
AZT+3TC
FTC+TDF
p=0.61
US RNA
log10 copies/µg total RNA
time ART, weeks
3TC+TDF
AZT+3TC
FTC+TDF
p=0.63
Total DNA
log10 copies/10^6 PBMC
time ART, weeks
3TC+TDF
AZT+3TC
FTC+TDF
p=0.46
Plasma viral load
log10 copies/mL
time ART, weeks
3TC+TDF
AZT+3TC
FTC+TDF
p=0.52

Figure S4

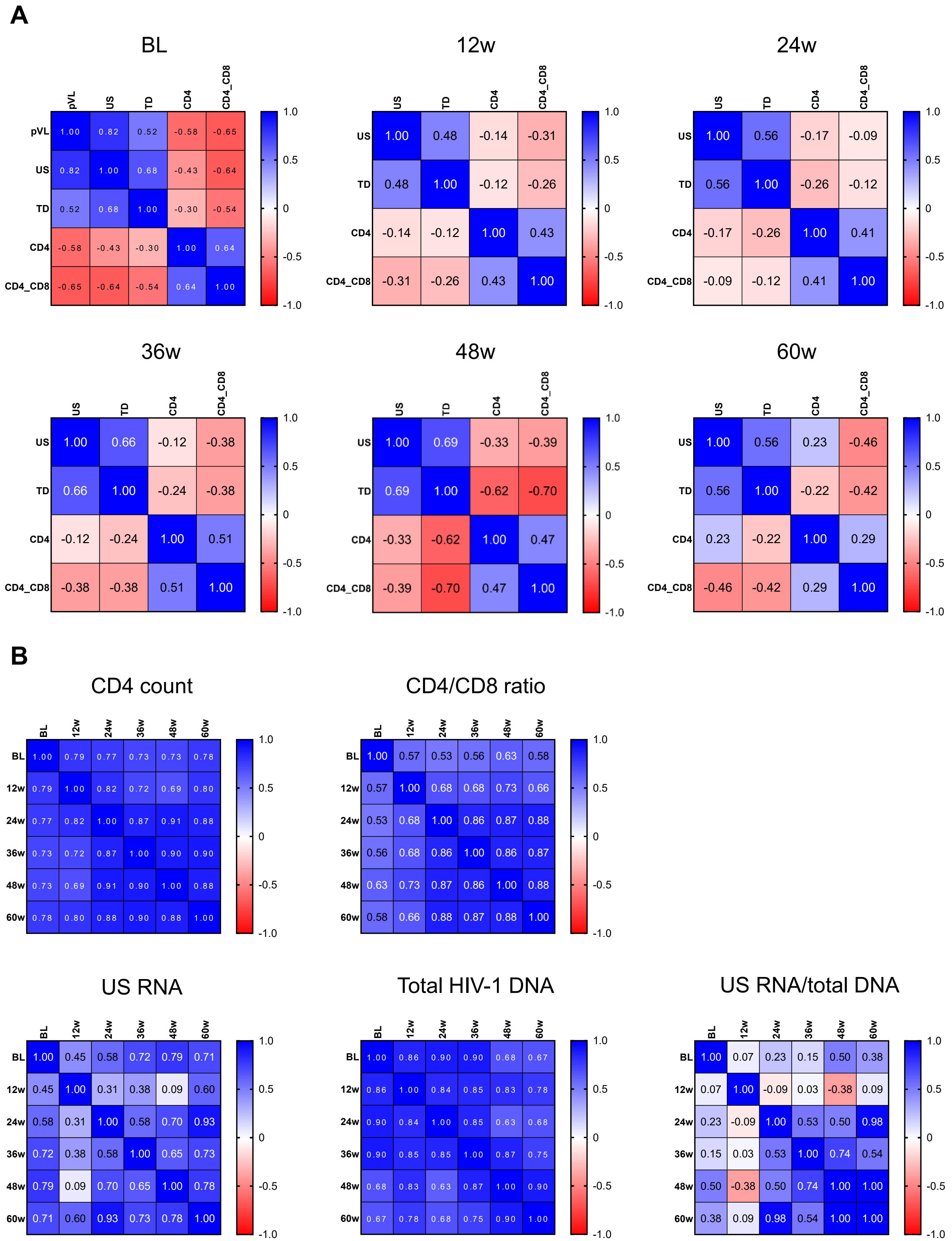
A
BL
pVL US TD CD4 CD4_CD8
pVL 1.00 0.82 0.52 -0.58 -0.65
US 0.82 1.00 0.68 -0.43 -0.64
TD 0.52 0.68 1.00 -0.30 -0.54
CD4 -0.58 -0.43 -0.30 1.00 0.64
CD4_CD8 -0.65 -0.64 -0.54 0.64 1.00
1.0 0.5 0 -0.5 -1.0
12w
US TD CD4 CD4_CD8
US 1.00 0.48 -0.14 -0.31
TD 0.48 1.00 -0.12 -0.26
CD4 -0.14 -0.12 1.00 0.43
CD4_CD8 -0.31 -0.26 0.43 1.00
1.0 0.5 0 -0.5 -1.0
24w
US TD CD4 CD4_CD8
US 1.00 0.56 -0.17 -0.09
TD 0.56 1.00 -0.26 -0.12
CD4 -0.17 -0.26 1.00 0.41
CD4_CD8 -0.09 -0.12 0.41 1.00
1.0 0.5 0 -0.5 -1.0
36w
US TD CD4 CD4_CD8
US 1.00 0.66 -0.12 -0.38
TD 0.66 1.00 -0.24 -0.38
CD4 -0.12 -0.24 1.00 0.51
CD4_CD8 -0.38 -0.38 0.51 1.00
1.0 0.5 0 -0.5 -1.0
48w
US TD CD4 CD4_CD8
US 1.00 0.69 -0.33 -0.39
TD 0.69 1.00 -0.62 -0.70
CD4 -0.33 -0.62 1.00 0.47
CD4_CD8 -0.39 -0.70 0.47 1.00
1.0 0.5 0 -0.5 -1.0
60w
US TD CD4 CD4_CD8
US 1.00 0.56 0.23 -0.46
TD 0.56 1.00 -0.22 -0.42
CD4 0.23 -0.22 1.00 0.29
CD4_CD8 -0.46 -0.42 0.29 1.00
1.0 0.5 0 -0.5 -1.0
B
CD4 count
BL 12w 24w 36w 48w 60w
BL 1.00 0.79 0.77 0.73 0.73 0.78
12w 0.79 1.00 0.82 0.72 0.69 0.80
24w 0.77 0.82 1.00 0.87 0.91 0.88
36w 0.73 0.72 0.87 1.00 0.90 0.90
48w 0.73 0.69 0.91 0.90 1.00 0.88
60w 0.78 0.80 0.88 0.90 0.88 1.00
1.0 0.5 0 -0.5 -1.0
CD4/CD8 ratio
BL 12w 24w 36w 48w 60w
BL 1.00 0.57 0.53 0.56 0.63 0.58
12w 0.57 1.00 0.68 0.68 0.73 0.66
24w 0.53 0.68 1.00 0.86 0.87 0.88
36w 0.56 0.68 0.86 1.00 0.86 0.87
48w 0.63 0.73 0.87 0.86 1.00 0.88
60w 0.58 0.66 0.88 0.87 0.88 1.00
1.0 0.5 0 -0.5 -1.0
US RNA
BL 12w 24w 36w 48w 60w
BL 1.00 0.45 0.58 0.72 0.79 0.71
12w 0.45 1.00 0.31 0.38 0.09 0.60
24w 0.58 0.31 1.00 0.58 0.70 0.93
36w 0.72 0.38 0.58 1.00 0.65 0.73
48w 0.79 0.09 0.70 0.65 1.00 0.78
60w 0.71 0.60 0.93 0.73 0.78 1.00
1.0 0.5 0 -0.5 -1.0
Total HIV-1 DNA
BL 12w 24w 36w 48w 60w
BL 1.00 0.86 0.90 0.90 0.68 0.67
12w 0.86 1.00 0.84 0.85 0.83 0.78
24w 0.90 0.84 1.00 0.85 0.63 0.68
36w 0.90 0.85 0.85 1.00 0.87 0.75
48w 0.68 0.83 0.63 0.87 1.00 0.90
60w 0.67 0.78 0.68 0.75 0.90 1.00
1.0 0.5 0 -0.5 -1.0
US RNA/total DNA
BL 12w 24w 36w 48w 60w
BL 1.00 0.07 0.23 0.15 0.50 0.38
12w 0.07 1.00 -0.09 0.03 -0.38 0.09
24w 0.23 -0.09 1.00 0.53 0.50 0.98
36w 0.15 0.03 0.53 1.00 0.74 0.54
48w 0.50 -0.38 0.50 0.74 1.00 1.00
60w 0.38 0.09 0.98 0.54 1.00 1.00
1.0 0.5 0 -0.5 -1.0

Figure S5

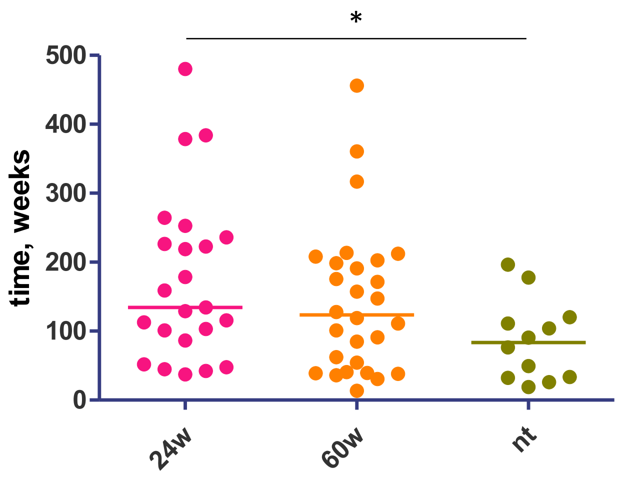

Kruskal-Wallis p=0.039

Figure S6

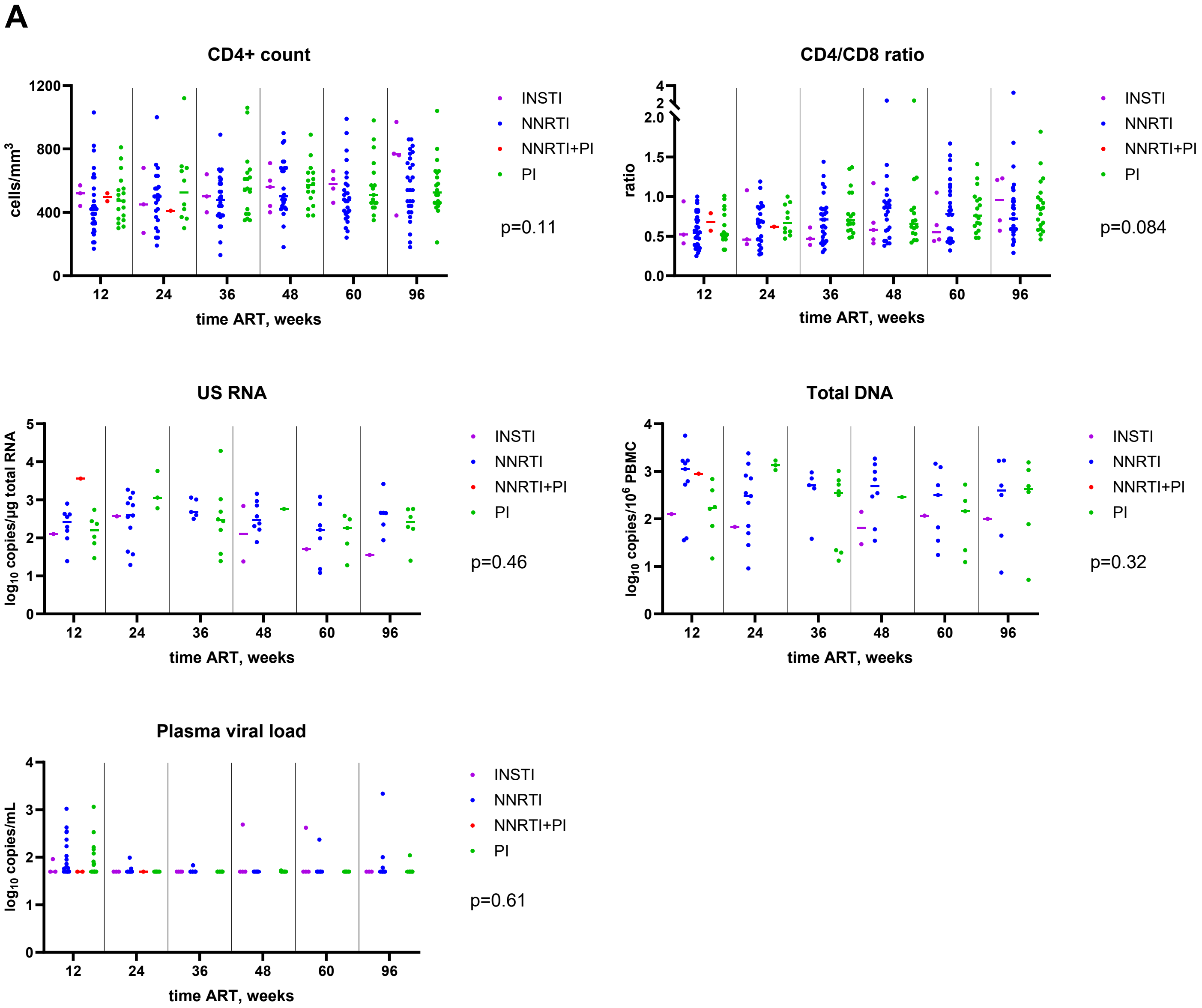
A
CD4+ count
cells/mm3
time ART, weeks
12 24 36 48 60 96
INSTI
NNRTI
NNRTI+PI
PI
p=0.11
CD4/CD8 ratio
ratio
p=0.084
US RNA
log10 copies/µg total RNA
p=0.46
Total DNA
log10 copies/10^6 PBMC
p=0.32
Plasma viral load
log10 copies/mL
p=0.61

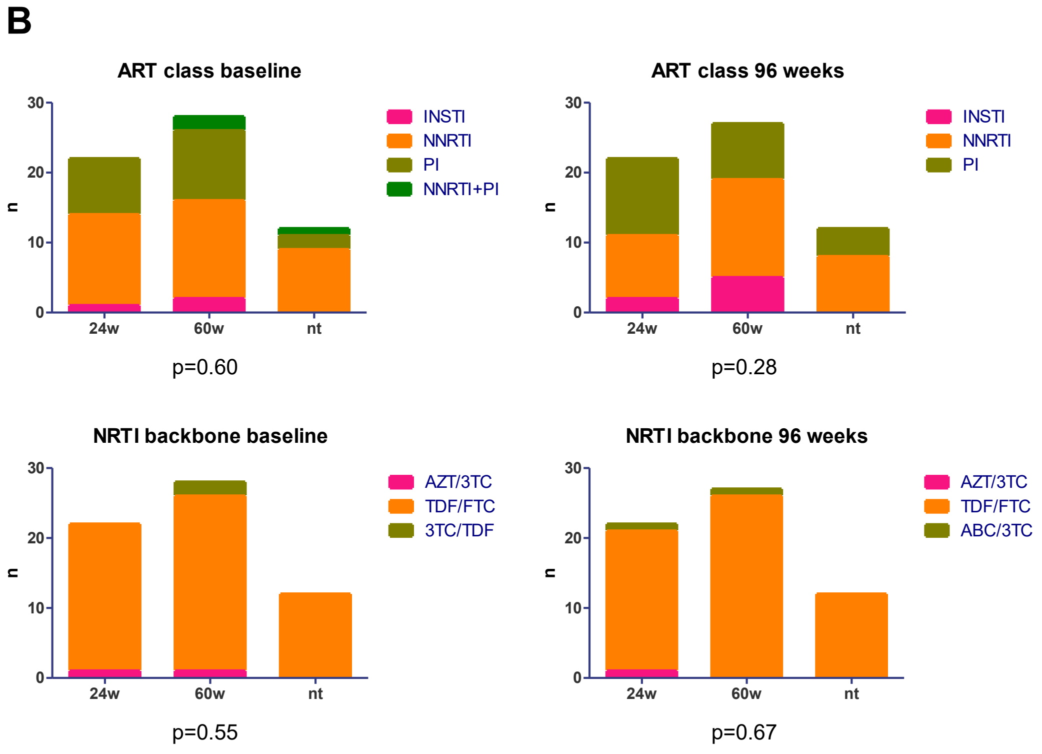
B
ART class baseline
ART class 96 weeks
n
24w
60w
nt
INSTI
NNRTI
PI
NNRTI+PI
p=0.60
p=0.28
NRTI backbone baseline
NRTI backbone 96 weeks
AZT/3TC
TDF/FTC
3TC/TDF
ABC/3TC
p=0.55
p=0.67

Figure S7

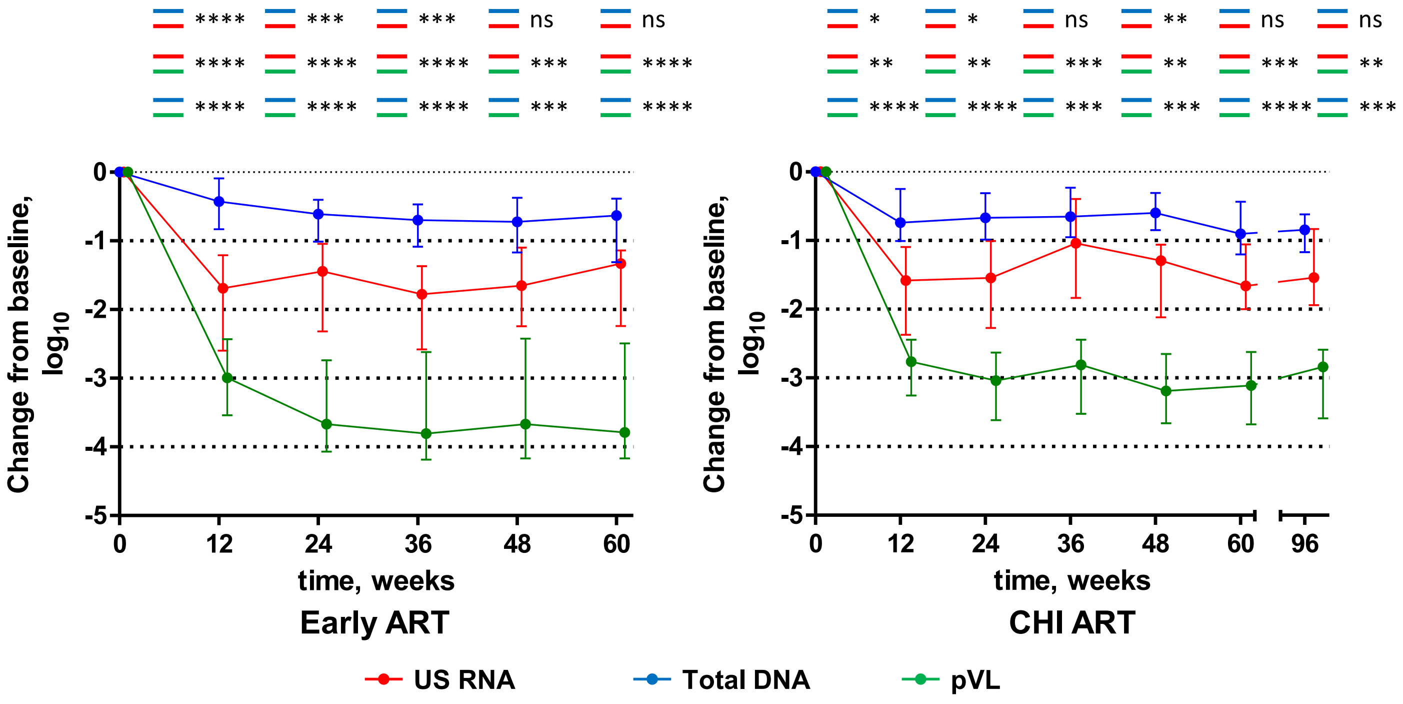

**** *** *** ns ns
**** **** **** *** ****
**** **** **** *** ****
* * ns ** ns ns
** ** *** ** *** **
**** **** *** *** **** ***
Change from baseline, log10
0
-1
-2
-3
-4
-5
0 12 24 36 48 60
time, weeks
Early ART
0 12 24 36 48 60 96
time, weeks
CHI ART
US RNA
Total DNA
pVL

Figure S8

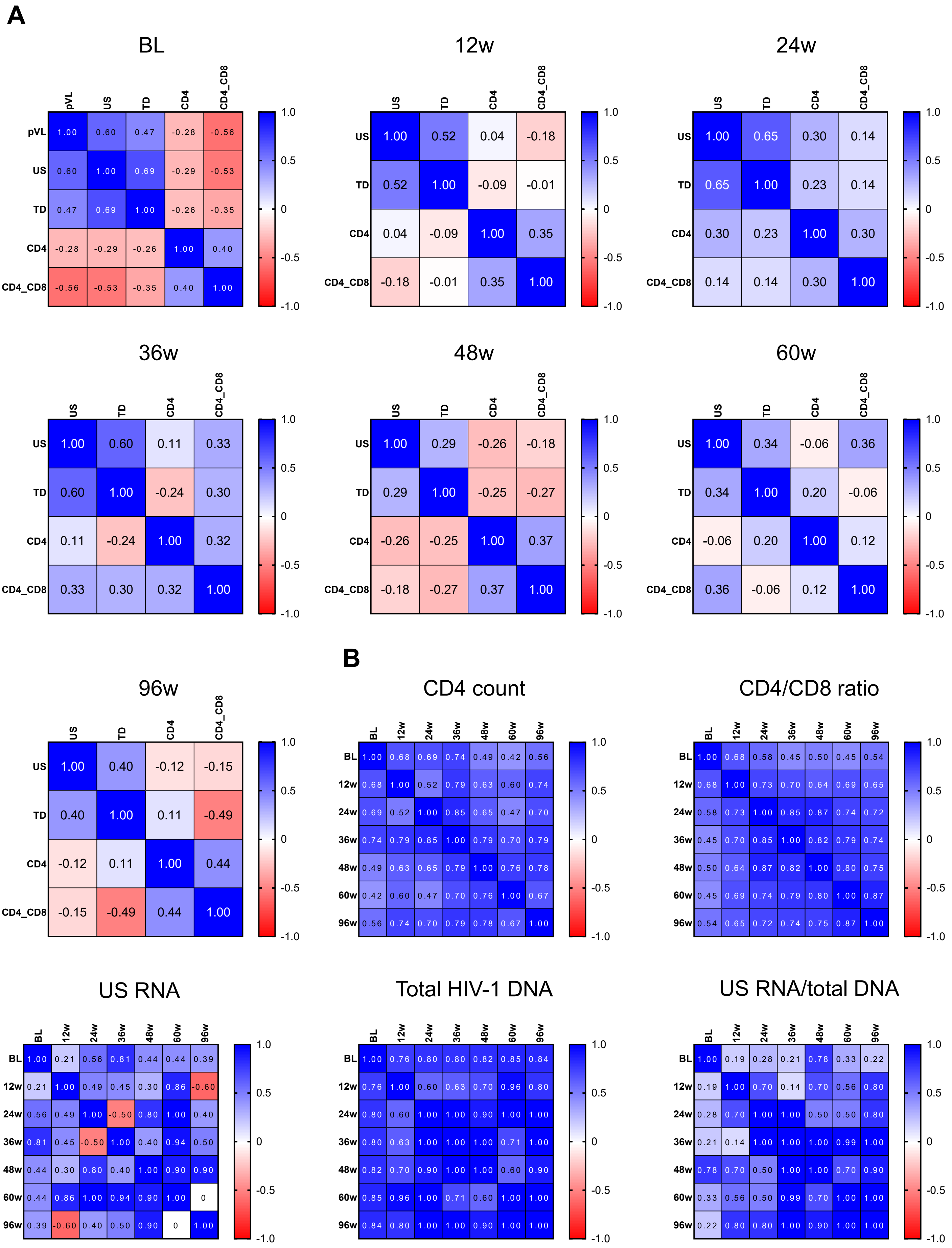
A
BL
pVL US TD CD4 CD4_CD8
pVL 1.00 0.60 0.47 -0.28 -0.56
US 0.60 1.00 0.69 -0.29 -0.53
TD 0.47 0.69 1.00 -0.26 -0.35
CD4 -0.28 -0.29 -0.26 1.00 0.40
CD4_CD8 -0.56 -0.53 -0.35 0.40 1.00
12w
US TD CD4 CD4_CD8
US 1.00 0.52 0.04 -0.18
TD 0.52 1.00 -0.09 -0.01
CD4 0.04 -0.09 1.00 0.35
CD4_CD8 -0.18 -0.01 0.35 1.00
24w
US TD CD4 CD4_CD8
US 1.00 0.65 0.30 0.14
TD 0.65 1.00 0.23 0.14
CD4 0.30 0.23 1.00 0.30
CD4_CD8 0.14 0.14 0.30 1.00
36w
US TD CD4 CD4_CD8
US 1.00 0.60 0.11 0.33
TD 0.60 1.00 -0.24 0.30
CD4 0.11 -0.24 1.00 0.32
CD4_CD8 0.33 0.30 0.32 1.00
48w
US TD CD4 CD4_CD8
US 1.00 0.29 -0.26 -0.18
TD 0.29 1.00 -0.25 -0.27
CD4 -0.26 -0.25 1.00 0.37
CD4_CD8 -0.18 -0.27 0.37 1.00
60w
US TD CD4 CD4_CD8
US 1.00 0.34 -0.06 0.36
TD 0.34 1.00 0.20 -0.06
CD4 -0.06 0.20 1.00 0.12
CD4_CD8 0.36 -0.06 0.12 1.00
96w
US TD CD4 CD4_CD8
US 1.00 0.40 -0.12 -0.15
TD 0.40 1.00 0.11 -0.49
CD4 -0.12 0.11 1.00 0.44
CD4_CD8 -0.15 -0.49 0.44 1.00
B
CD4 count
BL 12w 24w 36w 48w 60w 96w
BL 1.00 0.68 0.69 0.74 0.49 0.42 0.56
12w 0.68 1.00 0.52 0.79 0.63 0.60 0.74
24w 0.69 0.52 1.00 0.85 0.65 0.47 0.70
36w 0.74 0.79 0.85 1.00 0.79 0.70 0.79
48w 0.49 0.63 0.65 0.79 1.00 0.76 0.78
60w 0.42 0.60 0.47 0.70 0.76 1.00 0.67
96w 0.56 0.74 0.70 0.79 0.78 0.67 1.00
CD4/CD8 ratio
BL 12w 24w 36w 48w 60w 96w
BL 1.00 0.68 0.58 0.45 0.50 0.45 0.54
12w 0.68 1.00 0.73 0.70 0.64 0.69 0.65
24w 0.58 0.73 1.00 0.85 0.87 0.74 0.72
36w 0.45 0.70 0.85 1.00 0.82 0.79 0.74
48w 0.50 0.64 0.87 0.82 1.00 0.80 0.75
60w 0.45 0.69 0.74 0.79 0.80 1.00 0.87
96w 0.54 0.65 0.72 0.74 0.75 0.87 1.00
US RNA
BL 12w 24w 36w 48w 60w 96w
BL 1.00 0.21 0.56 0.81 0.44 0.44 0.39
12w 0.21 1.00 0.49 0.45 0.30 0.86 -0.60
24w 0.56 0.49 1.00 -0.50 0.80 1.00 0.40
36w 0.81 0.45 -0.50 1.00 0.40 0.94 0.50
48w 0.44 0.30 0.80 0.40 1.00 0.90 0.90
60w 0.44 0.86 1.00 0.94 0.90 1.00 0
96w 0.39 -0.60 0.40 0.50 0.90 0 1.00
Total HIV-1 DNA
BL 12w 24w 36w 48w 60w 96w
BL 1.00 0.76 0.80 0.80 0.82 0.85 0.84
12w 0.76 1.00 0.60 0.63 0.70 0.96 0.80
24w 0.80 0.60 1.00 1.00 0.90 1.00 1.00
36w 0.80 0.63 1.00 1.00 1.00 0.71 1.00
48w 0.82 0.70 0.90 1.00 1.00 0.60 0.90
60w 0.85 0.96 1.00 0.71 0.60 1.00 1.00
96w 0.84 0.80 1.00 1.00 0.90 1.00 1.00
US RNA/total DNA
BL 12w 24w 36w 48w 60w 96w
BL 1.00 0.19 0.28 0.21 0.78 0.33 0.22
12w 0.19 1.00 0.70 0.14 0.70 0.56 0.80
24w 0.28 0.70 1.00 1.00 0.50 0.50 0.80
36w 0.21 0.14 1.00 1.00 1.00 0.99 1.00
48w 0.78 0.70 0.50 1.00 1.00 0.70 0.90
60w 0.33 0.56 0.50 0.99 0.70 1.00 1.00
96w 0.22 0.80 0.80 1.00 0.90 1.00 1.00
1.0 0.5 0 -0.5 -1.0

Figure S9

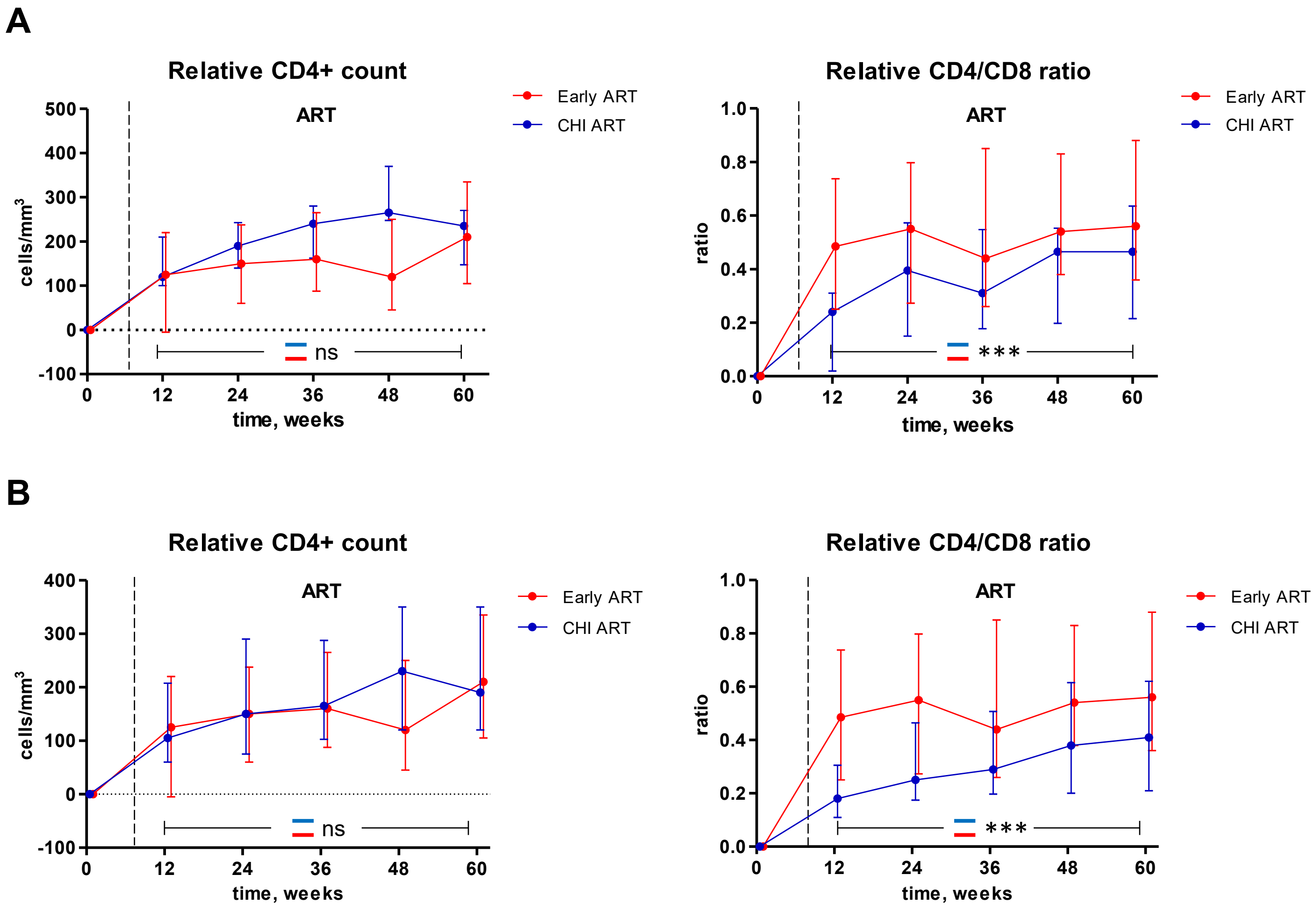
A
Relative CD4+ count
ART
cells/mm3
time, weeks
ns
Early ART
CHI ART
Relative CD4/CD8 ratio
ART
ratio
time, weeks
***
Early ART
CHI ART
B
Relative CD4+ count
ART
cells/mm3
time, weeks
ns
Early ART
CHI ART
Relative CD4/CD8 ratio
ART
ratio
time, weeks
***
Early ART
CHI ART

Figure S10

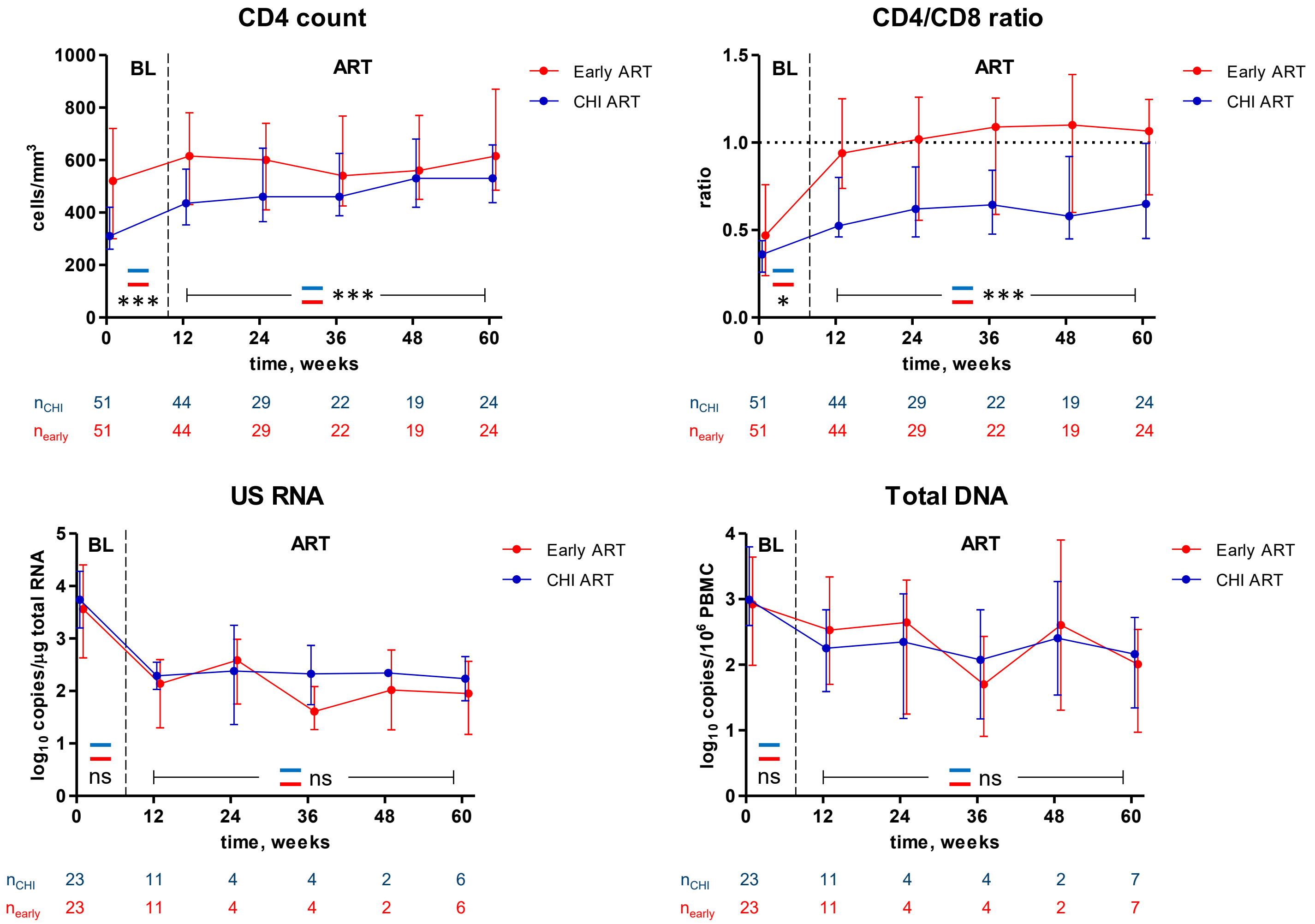
CD4 count
cells/mm3
BL
ART
***
***
time, weeks
nCHI 51 44 29 22 19 24
nearly 51 44 29 22 19 24
Early ART
CHI ART
CD4/CD8 ratio
ratio
BL
ART
*
***
time, weeks
nCHI 51 44 29 22 19 24
nearly 51 44 29 22 19 24
Early ART
CHI ART
US RNA
log10 copies/μg total RNA
BL
ART
ns
ns
time, weeks
nCHI 23 11 4 4 2 6
nearly 23 11 4 4 2 6
Early ART
CHI ART
Total DNA
log10 copies/10^6 PBMC
BL
ART
ns
ns
time, weeks
nCHI 23 11 4 4 2 7
nearly 23 11 4 4 2 7
Early ART
CHI ART

Figure S11

Early ART

	CD4+ proliferation	CD8+ proliferation	CD4+ reactivity	CD8+ reactivity	IFN-γ release
Intact HIV DNA	-0.12	-0.12	0.04	-0.37	-0.41
3' defective HIV-1 DNA	-0.15	-0.28	0.08	-0.03	-0.28
5' defective HIV-1 DNA	-0.36	-0.36	0.04	-0.27	-0.56

CHI ART

	CD4+ proliferation	CD8+ proliferation	CD4+ reactivity	CD8+ reactivity	IFN-γ release
Intact HIV DNA	-0.37	-0.54	0.13	-0.49	-0.59
3' defective HIV-1 DNA	-0.30	-0.39	-0.08	0.05	-0.09
5' defective HIV-1 DNA	-0.20	-0.44	0.17	-0.37	-0.44

Figure S12

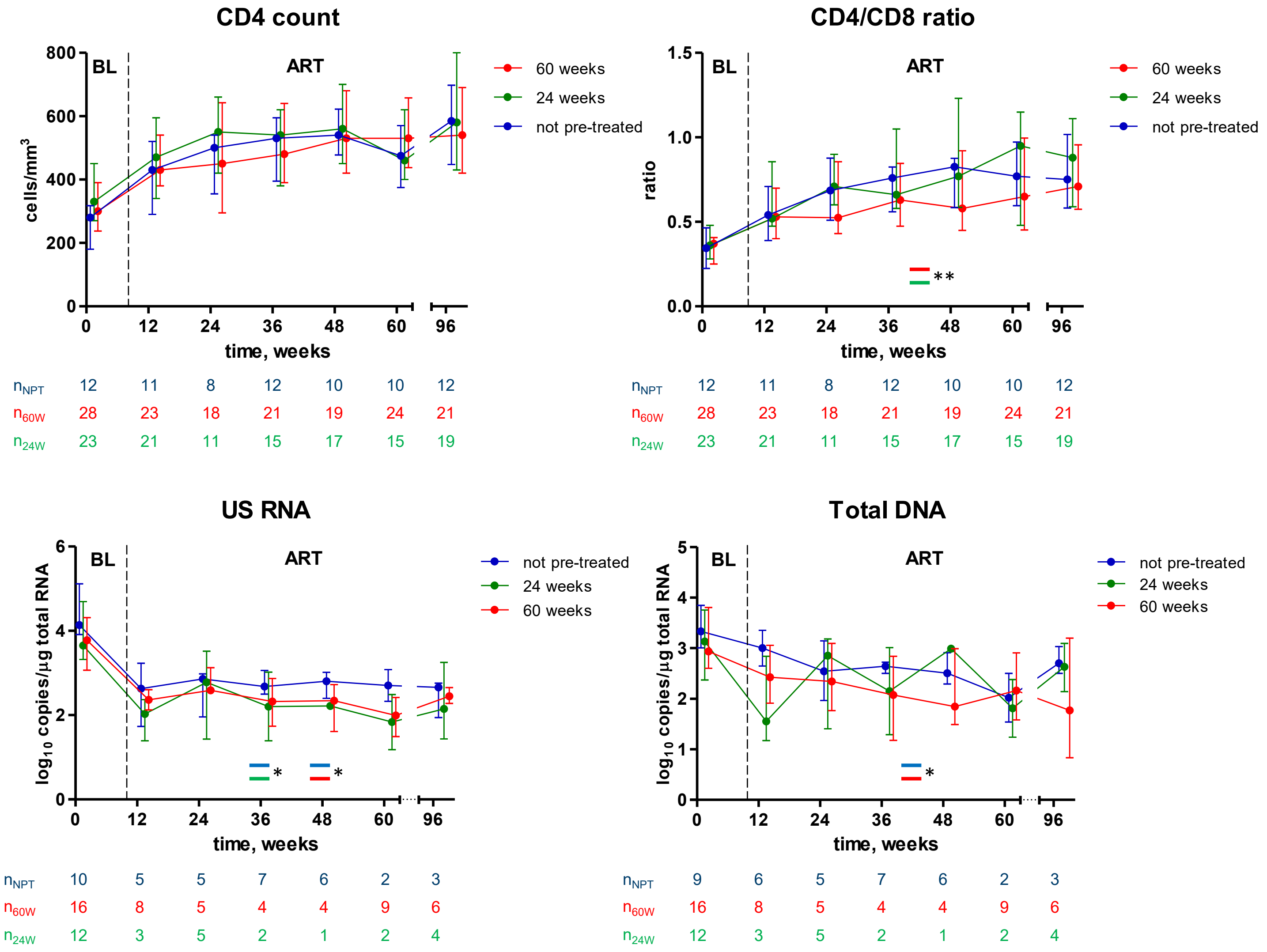
CD4 count
cells/mm³
BL
ART
60 weeks
24 weeks
not pre-treated
800 600 400 200 0
0 12 24 36 48 60 96
time, weeks
nNPT 12 11 8 12 10 10 12
n60W 28 23 18 21 19 24 21
n24W 23 21 11 15 17 15 19
CD4/CD8 ratio
ratio
BL
ART
60 weeks
24 weeks
not pre-treated
1.5 1.0 0.5 0.0
**
0 12 24 36 48 60 96
time, weeks
nNPT 12 11 8 12 10 10 12
n60W 28 23 18 21 19 24 21
n24W 23 21 11 15 17 15 19
US RNA
log10 copies/µg total RNA
BL
ART
not pre-treated
24 weeks
60 weeks
6 4 2 0
*
*
0 12 24 36 48 60 96
time, weeks
nNPT 10 5 5 7 6 2 3
n60W 16 8 5 4 4 9 6
n24W 12 3 5 2 1 2 4
Total DNA
log10 copies/µg total RNA
BL
ART
not pre-treated
24 weeks
60 weeks
5 4 3 2 1 0
*
0 12 24 36 48 60 96
time, weeks
nNPT 9 6 5 7 6 2 3
n60W 16 8 5 4 4 9 6
n24W 12 3 5 2 1 2 4

Figure S13

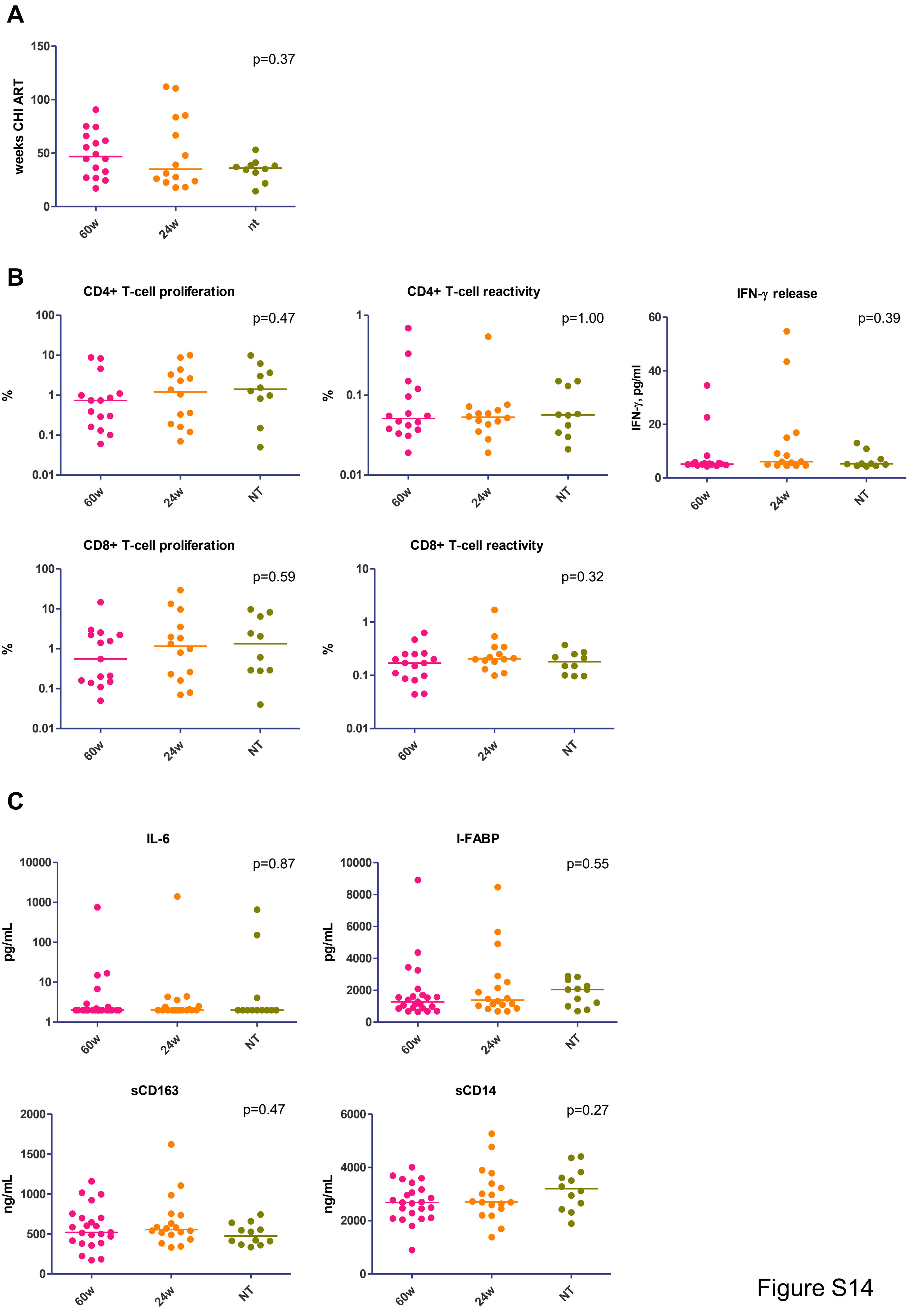
A
weeks CHI ART
p=0.37
60w
24w
nt
B
CD4+ T-cell proliferation
p=0.47
CD4+ T-cell reactivity
p=1.00
IFN-γ release
IFN-γ, pg/ml
p=0.39
CD8+ T-cell proliferation
p=0.59
CD8+ T-cell reactivity
p=0.32
%
60w
24w
NT
C
IL-6
pg/mL
p=0.87
I-FABP
p=0.55
sCD163
ng/mL
p=0.47
sCD14
p=0.27

Figure S14

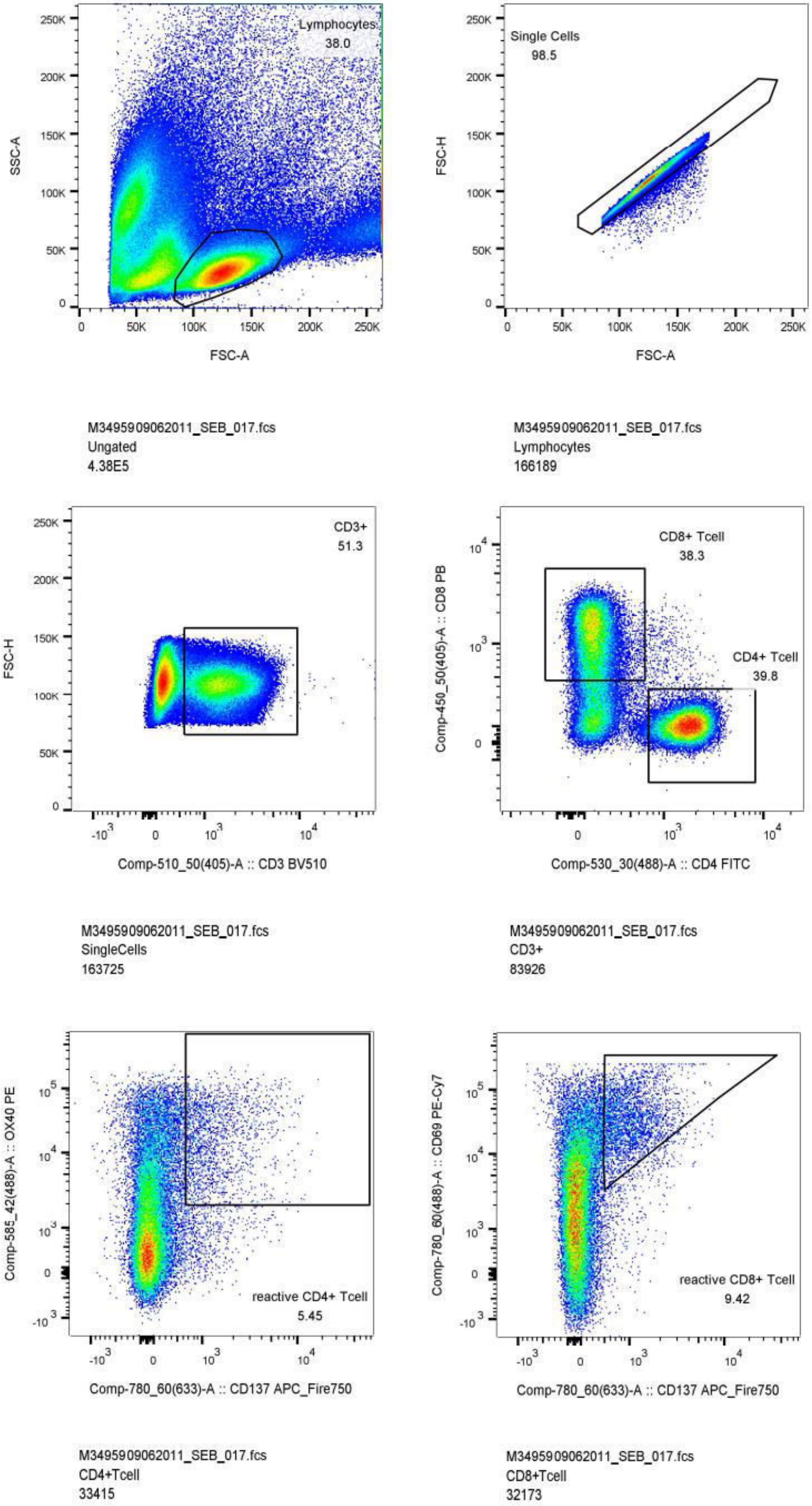
Lymphocytes
38.0
SSC-A
FSC-A
M3495909062011_SEB_017.fcs
Ungated
4.38E5
Single Cells
98.5
FSC-H
FSC-A
M3495909062011_SEB_017.fcs
Lymphocytes
166189
CD3+
51.3
FSC-H
Comp-510_50(405)-A :: CD3 BV510
M3495909062011_SEB_017.fcs
SingleCells
163725
CD8+ Tcell
38.3
CD4+ Tcell
39.8
Comp-450_50(405)-A :: CD8 PB
Comp-530_30(488)-A :: CD4 FITC
M3495909062011_SEB_017.fcs
CD3+
83926
reactive CD4+ Tcell
5.45
Comp-585_42(488)-A :: OX40 PE
Comp-780_60(633)-A :: CD137 APC_Fire750
M3495909062011_SEB_017.fcs
CD4+Tcell
33415
reactive CD8+ Tcell
9.42
Comp-780_60(488)-A :: CD69 PE-Cy7
Comp-780_60(633)-A :: CD137 APC_Fire750
M3495909062011_SEB_017.fcs
CD8+Tcell
32173

Figure S15

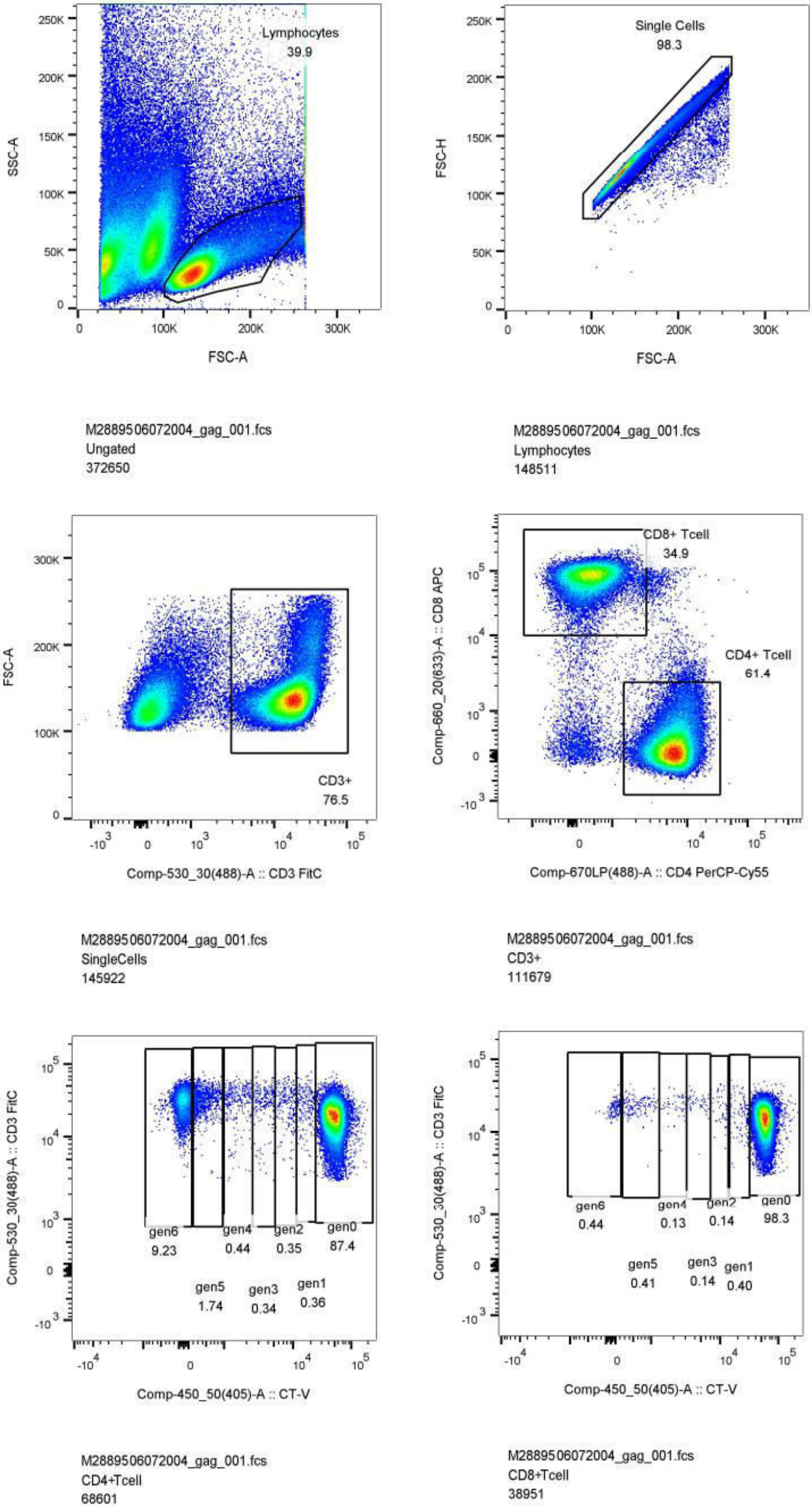
Lymphocytes
39.9
SSC-A
FSC-A
M2889506072004_gag_001.fcs
Ungated
372650
Single Cells
98.3
FSC-H
FSC-A
M2889506072004_gag_001.fcs
Lymphocytes
148511
CD3+
76.5
FSC-A
Comp-530_30(488)-A :: CD3 FitC
M2889506072004_gag_001.fcs
SingleCells
145922
CD8+ Tcell
34.9
CD4+ Tcell
61.4
Comp-660_20(633)-A :: CD8 APC
Comp-670LP(488)-A :: CD4 PerCP-Cy55
M2889506072004_gag_001.fcs
CD3+
111679
gen6 9.23
gen5 1.74
gen4 0.44
gen3 0.34
gen2 0.35
gen1 0.36
gen0 87.4
Comp-530_30(488)-A :: CD3 FitC
Comp-450_50(405)-A :: CT-V
M2889506072004_gag_001.fcs
CD4+Tcell
68601
gen6 0.44
gen5 0.41
gen4 0.13
gen3 0.14
gen2 0.14
gen1 0.40
gen0 98.3
Comp-530_30(488)-A :: CD3 FitC
Comp-450_50(405)-A :: CT-V
M2889506072004_gag_001.fcs
CD8+Tcell
38951

Figure S16

Supplementary figure legends

Figure S1. Levels of the measured parameters during early ART in the 24-week and 60-week arms. Participants are color-coded. For plasma viral load (VL), limit of detection of the commercial assays (50 copies/mL) is shown with a dashed line. Parameters were compared between 24-week and 60-week arms at baseline (BL), 12 weeks, and 24 weeks ART using Mann-Whitney tests. Data points represent individual participants (n=23 for 24-week arm, n=29 for 60-week arm). Source data are provided as a Source Data file.

Figure S2. P values of pairwise comparisons between the parameter values at different time points of early (A) and CHI (B) ART, adjusted to account for multiple comparisons. Only significant p values are shown.

Figure S3. Relative increases from baseline of CD4+ counts and CD4/CD8 ratios during early (A) and CHI (B) ART. Participants are color-coded. Data points represent individual participants (n=52 for panel A, n=64 for panel B). Source data are provided as a Source Data file.

Figure S4. Levels of HIV-1 persistence and immunological response parameters, stratified according to the ART class (A) or nucleoside analogue reverse transcriptase inhibitor (NRTI) backbone (B) that the participants received at every time point of early ART. Repeated-measures mixed-effects analyses and Mann-Whitney tests (to compare parameters at individual time points) were used to calculate the statistical significance. **, $0.001<p<0.01$; *, $0.01<p<0.05$. Only significant p values from the Mann-Whitney tests are shown. P values of the mixed-effects analyses are indicated in every graph. Exact p values from the Mann-Whitney tests: ART class, total DNA, 12 weeks: p=0.0057, ART class, plasma VL, 12 weeks: p=0.028. Source data are provided as a Source Data file.

Figure S5. Pairwise correlations between parameters per time point (A) and between time points per parameter (B) during early ART. Spearman *rho* values are shown.

Figure S6. Times without treatment between the interruption of early ART (for 60-week and 24-week arms) or randomization (for the no-treatment arm) and the start of CHI ART. Times without treatment were compared between the arms using Kruskal-Wallis test with Dunn's multiple comparison post-tests. The Kruskal-Wallis p value and the Dunn's post-test that was significant are shown. *, 0.01<p<0.05. n=23 (24-week arm), n=28 (60-week arm), n=12 (no-treatment arm). Source data are provided as a Source Data file. nt, no-treatment arm.

Figure S7. (A) Levels of HIV-1 persistence and immunological response parameters, stratified according to the ART class that the participants received at every time point of CHI ART. Repeated-measures mixed-effects analyses were used to calculate the statistical significance. (B) ART classes and NRTI backbones at baseline and at 96 weeks of CHI ART are shown for the 24-week, 60-week, and no-treatment study arms. Chi-squared tests were used to calculate the statistical significance. For all panels, source data are provided as a Source Data file. nt, no-treatment arm.

Figure S8. Relative changes of virological biomarkers from baseline at early and CHI ART. Log-transformed changes from baseline of US RNA, total DNA and plasma viral load (pVL) are shown. Participant numbers: n=52 for early ART, n=39 for CHI ART. Changes from baseline were compared between the parameters using repeated-measures mixed-effects modelling. ****, p<0.0001; ***, 0.0001<p<0.001; **, 0.001<p<0.01; *, 0.01<p<0.05; ns, not significant. Source data are provided as a Source Data file.

Figure S9. Pairwise correlations between parameters per time point (A) and between time points per parameter (B) during CHI ART. Spearman *rho* values are shown.

Figure S10. Comparisons of relative increases from baseline of CD4+ counts and CD4/CD8 ratios between early (red) and CHI (blue) ART in different (A) and in the same (B) participants. Participant numbers: n=52 for early ART, n=12 for CHI ART (A), n=52 for early ART, n=52 for CHI ART (B). Repeated-measures mixed-effects modelling was used for comparisons. ***, p<0.001; ns, not significant. Exact p values are as follows: CD4 count: p=0.097, CD4/CD8 ratio: $p=5.9*10^{-4}$ (A); CD4 count: p=0.093, CD4/CD8 ratio: $p=1.1*10^{-10}$ (B). Source data are provided as a Source Data file.

Figure S11. Sensitivity analysis for the comparisons between early (red) and CHI ART (blue) periods in the same participants. For each time point, only paired early and CHI ART measurements were included in the analysis. Baseline parameters were compared using Mann-Whitney tests and parameters measured under ART were compared using repeated-measures mixed-effects modelling. Numbers of participants per time point are indicated below the graphs. ***, p<0.001; ns, not significant. Exact p values are as follows. CD4 count: p<0.0001 (baseline), $p=9.5*10^{-5}$ (ART). CD4/CD8 ratio: p=0.035 (baseline), $p=2.7*10^{-16}$ (ART). US RNA: p=0.52 (baseline), p=0.13 (ART). Total DNA: p=0.19 (baseline), p=0.80 (ART). Source data are provided as a Source Data file.

Figure S12. *Rho* values of the Spearman correlations between intact, 3' defective, or 5' defective HIV-1 DNA levels and functional HIV-specific T-cell responses at early (A) and CHI (B) ART. Heat maps of the correlations are shown.

Figure S13. Comparisons during CHI ART between participants who were pre-treated with 60 weeks (red), 24 weeks (green), or not pre-treated (blue) with early ART. Parameters were compared using repeated-measures mixed-effects modelling. Numbers of participants per time point are indicated below the graphs. Only significant differences are shown. **, 0.001<p<0.01; *, 0.01<p<0.05. Exact p values are as follows. CD4/CD8 ratio: p=0.0086 (ART,

24-week arm vs. 60-week arm). US RNA: p=0.011 (ART, 24-week arm vs. no-treatment arm), p=0.017 (ART, 60-week arm vs. no-treatment arm). Total DNA: p=0.022 (ART, 60-week arm vs. no-treatment arm). Source data are provided as a Source Data file.

Figure S14. (A) Sampling times for the analyses of functional HIV-specific T-cell responses at CHI ART. n=14 (24-week arm), n=16 (60-week arm), n=10 (no-treatment arm). (B) Functional HIV-specific CD4+ and CD8+ T-cell responses at CHI ART for the 24-week, 60-week, and no-treatment study arms. n=14 (24-week arm), n=16 (60-week arm), n=10 (no-treatment arm). (C) Plasma biomarkers of systemic inflammation, intestinal damage, and monocyte activation at CHI ART for the 24-week, 60-week, and no-treatment study arms. n=18 (24-week arm), n=23 (60-week arm), n=12 (no-treatment arm) For all panels: Kruskal-Wallis tests were used to calculate the statistical significance. For all panels, source data are provided as a Source Data file.

Figure S15. Example of the gating strategy for the AIM assay.

Figure S16. Example of the gating strategy for the proliferation assay.

Table S1. Comparison of two-phase slopes[a].

		**CD4+ count, cells/mm^3/week**			**CD4/CD8 ratio, per week**			**US RNA, log_{10}/week**			**Total DNA, log_{10}/week**		
Early ART vs. CHI ART (no-treatment arm)		Slope early ART	Slope CHI ART	p[b]	Slope early ART	Slope CHI ART	p	Slope early ART	Slope CHI ART	p	Slope early ART	Slope CHI ART	p
	Phase I	10.68<br>(3.14-18.22)[c]<br>**p=0.0059[d]**	13.18<br>(4.90-21.46)<br>**p=0.0023**	0.76	0.043<br>(0.030-0.056)<br>**p<0.0001**	0.020<br>(0.005-0.035)<br>**p=0.0089**	0.12	-0.14<br>(-0.17--0.12)<br>**p<0.0001**	-0.15<br>(-0.20--0.10)<br>**p<0.0001**	0.92	-0.035<br>(-0.062--0.007)<br>**p=0.013**	-0.039<br>(-0.080-0.002)<br>p=0.061	0.89
	Phase II	0.25<br>(-1.92-2.43)<br>p=0.82	1.48<br>(-0.70-3.65)<br>p=0.18	0.58	0.003<br>(-0.001-0.007)<br>p=0.12	0.005<br>(0.001-0.009)<br>**p=0.0078**	0.53	0.003<br>(-0.005 - 0.011)<br>p=0.43	0.006<br>(-0.011-0.022)<br>p=0.48	0.83	-0.014<br>(-0.022--0.006)<br>**p=0.0011**	-0.015<br>(-0.028--0.001)<br>**p=0.036**	0.97
Early ART vs. CHI ART (same participants)		Slope early ART	Slope CHI ART	p	Slope early ART	Slope CHI ART	p	Slope early ART	Slope CHI ART	p	Slope early ART	Slope CHI ART	p
	Phase I	10.69<br>(3.14-18.22)<br>**p=0.0059**	10.67<br>(5.28-16.06)<br>**p=0.0001**	1.00	0.043<br>(0.030-0.056)<br>**p<0.0001**	0.019<br>(0.010-0.028)<br>**p<0.0001**	**0.0033**	-0.14<br>(-0.18--0.10)<br>**p<0.0001**	-0.11<br>(-0.14--0.07)<br>**p<0.0001**	0.22	-0.032<br>(-0.074-0.011)<br>p=0.14	-0.058<br>(-0.097--0.018)<br>**p=0.0052**	0.39
	Phase II	0.25<br>(-1.92-2.43)<br>p=0.82	1.69<br>(0.24-3.13)<br>**p=0.023**	0.28	0.003<br>(-0.001-0.007)<br>p=0.12	0.005<br>(0.002-0.007)<br>**p=0.0002**	0.47	0.002<br>(-0.009-0.013)<br>p=0.74	-0.008<br>(-0.020-0.003)<br>p=0.15	0.21	-0.012<br>(-0.024-0.001)<br>p=0.073	-0.003<br>(-0.016-0.010)<br>p=0.62	0.38
CHI ART (pre-treated vs. not pre-treated)		Slope pre-treated	Slope not pre-treated	p[e]	Slope pre-treated	Slope not pre-treated	p	Slope pre-treated	Slope not pre-treated	p	Slope pre-treated	Slope not pre-treated	p
	Phase I	11.52<br>(6.35-16.70)<br>**p<0.0001**	13.43<br>(5.43-21.42)<br>**p=0.0013**	0.74	0.021<br>(0.012-0.030)<br>**p<0.0001**	0.024<br>(0.008-0.040)<br>**p=0.0037**	0.77	-0.12<br>(-0.15--0.08)<br>**p<0.0001**	-0.14<br>(-0.18--0.09)<br>**p<0.0001**	0.48	-0.060<br>(-0.097--0.022)<br>**p=0.0021**	-0.054<br>(-0.093--0.016)<br>**p=0.007**	0.86
	Phase II	1.14<br>(0.30-1.98)<br>**p=0.0086**	1.32<br>(0.10-2.54)<br>**p=0.034**	0.83	0.003<br>(0.002-0.005)<br>**p<0.0001**	0.003<br>(0.001-0.006)<br>**p=0.0099**	0.95	-0.001<br>(-0.008-0.005)<br>p=0.66	0.000<br>(-0.010-0.009)<br>p=0.92	0.88	-0.002<br>(-0.009-0.006)<br>p=0.67	-0.004<br>(-0.012-0.004)<br>p=0.36	0.74

[a] Calculated using a linear two-phase segmentation model with change points forced at 12 weeks ART.
[b] Comparison between slopes of early and CHI ART. Extra sum-of-squares F tests were used to analyse the data.
[c] 95% confidence intervals are shown.
[d] Comparison of the slope with zero. Extra sum-of-squares F tests were used to analyse the data.
[e] Comparison between slopes of pre-treated and not pre-treated. Extra sum-of-squares F tests were used to analyse the data.
