# Supplementary material for: Long-term effect of temporary ART initiated during primary HIV-1 infection on viral persistence
Source: Nat Commun. 2025 Jul 30;16:6989. doi: 10.1038/s41467-025-62362-0 (PMC12311192; doi:10.1038/s41467-025-62362-0)
Supplement: Supplementary file 3 — Reporting Summary [file 41467_2025_62362_MOESM3_ESM.pdf]

Corresponding author(s): A. O. Pasternak

Last updated by author(s): Jul 2, 2025

Reporting Summary

Nature Portfolio wishes to improve the reproducibility of the work that we publish. This form provides structure for consistency and transparency in reporting. For further information on Nature Portfolio policies, see our Editorial Policies and the Editorial Policy Checklist.

Statistics

For all statistical analyses, confirm that the following items are present in the figure legend, table legend, main text, or Methods section.

n/a	Confirmed	
☐	☒	The exact sample size (*n*) for each experimental group/condition, given as a discrete number and unit of measurement
☒	☐	A statement on whether measurements were taken from distinct samples or whether the same sample was measured repeatedly
☐	☒	The statistical test(s) used AND whether they are one- or two-sided<br>*Only common tests should be described solely by name; describe more complex techniques in the Methods section.*
☒	☐	A description of all covariates tested
☒	☐	A description of any assumptions or corrections, such as tests of normality and adjustment for multiple comparisons
☐	☒	A full description of the statistical parameters including central tendency (e.g. means) or other basic estimates (e.g. regression coefficient) AND variation (e.g. standard deviation) or associated estimates of uncertainty (e.g. confidence intervals)
☐	☒	For null hypothesis testing, the test statistic (e.g. *F*, *t*, *r*) with confidence intervals, effect sizes, degrees of freedom and *P* value noted<br>*Give P values as exact values whenever suitable.*
☒	☐	For Bayesian analysis, information on the choice of priors and Markov chain Monte Carlo settings
☒	☐	For hierarchical and complex designs, identification of the appropriate level for tests and full reporting of outcomes
☐	☒	Estimates of effect sizes (e.g. Cohen's *d*, Pearson's *r*), indicating how they were calculated

Our web collection on statistics for biologists contains articles on many of the points above.

Software and code

Policy information about availability of computer code

Data collection	Quantitative PCR data were collected using Applied Biosystems Sequence Detection Software (version 1.2.3). Droplet digital PCR data were collected using QuantaSoft (version 1.7.4).
Data analysis	Data were analysed using Prism 10.2.0 (GraphPad Software) and IBM SPSS Statistics (version 28.0.1.0). Fluorescent marker expression levels were analyzed using FlowJo version 10.8.1 (TreeStar, Ashland, OR, USA). For measurements of HIV-1 diversity, sequences were assembled using CodonCode Aligner (version 10.0.2) and screened G-to-A hypermutation using Hypermut 2.0 software (https://www.hiv.lanl.gov/content/sequence/HYPERMUT/hypermut.html). Maximum likelihood analyses and diversity calculations were performed using MEGA 7.0 software.

For manuscripts utilizing custom algorithms or software that are central to the research but not yet described in published literature, software must be made available to editors and reviewers. We strongly encourage code deposition in a community repository (e.g. GitHub). See the Nature Portfolio guidelines for submitting code & software for further information.

Data

Policy information about availability of data

All manuscripts must include a data availability statement. This statement should provide the following information, where applicable:

- Accession codes, unique identifiers, or web links for publicly available datasets
- A description of any restrictions on data availability
- For clinical datasets or third party data, please ensure that the statement adheres to our policy

All data are available in the main text, the supplementary materials, or the source data file.

Research involving human participants, their data, or biological material

Policy information about studies with human participants or human data. See also policy information about sex, gender (identity/presentation), and sexual orientation and race, ethnicity and racism.

Reporting on sex and gender	Of the 64 participants enrolled in this study, only three were assigned as female at birth, which precluded the data analysis by sex.
Reporting on race, ethnicity, or other socially relevant groupings	This study did not use the constructs of race and/or ethnicity.
Population characteristics	The vast majority of the participants were MSM residing in the Netherlands who were infected with HIV-1 subtype B. Median age at the study entry was 39.8 (IQR, 31.7-47.1) years.
Recruitment	All 64 participants of the Primo-SHM trial who completed the trial in the Academic Medical Center of the University of Amsterdam (AMC) were included in this study. Therefore, no self-selection bias or other biases were possible.
Ethics oversight	The study was approved by the AMC Medical Ethics Committee.

Note that full information on the approval of the study protocol must also be provided in the manuscript.

Field-specific reporting

Please select the one below that is the best fit for your research. If you are not sure, read the appropriate sections before making your selection.

[x] Life sciences   [ ] Behavioural & social sciences   [ ] Ecological, evolutionary & environmental sciences

For a reference copy of the document with all sections, see nature.com/documents/nr-reporting-summary-flat.pdf

Life sciences study design

All studies must disclose on these points even when the disclosure is negative.

Sample size	No sample size calculation was performed because all 64 participants of the Primo-SHM trial who completed the trial in the Academic Medical Center of the University of Amsterdam were included in this study.
Data exclusions	No data were excluded from the analysis.
Replication	All assays used in this study have been thoroughly validated previously for accuracy and reproducibility (e.g. PMID: 18463204, PMID: 24465831). Due to limited sample volumes, virological and immunological markers have been measured once per sample.
Randomization	The parent study (Primo-SHM trial) was a multicentre RCT comparing temporary early ART with no treatment during PHI. Participants were randomly assigned to receive no treatment or 24 or 60 weeks of ART (3-way randomization). If treatment was clinically indicated based on severe clinical symptoms or the participant insisted on starting early ART, subjects were randomized over the 2 treatment arms (2-way randomization). nvestigators of the present study used samples from all 64 participants of the parent study who completed the trial in the Academic Medical Center of the University of Amsterdam.
Blinding	For the parent study, the Dutch HIV Monitoring Foundation performed the randomization procedure, had no interaction with study participants, and was responsible for data management. Randomization results were sent by fax to the clinical investigators, who were unaware of the allocation procedure. Investigators of the present study used samples from all 64 participants of the parent study who completed the trial in the Academic Medical Center of the University of Amsterdam.

Reporting for specific materials, systems and methods

We require information from authors about some types of materials, experimental systems and methods used in many studies. Here, indicate whether each material, system or method listed is relevant to your study. If you are not sure if a list item applies to your research, read the appropriate section before selecting a response.

Materials & experimental systems

n/a	Involved in the study
	☒ Antibodies
☒	Eukaryotic cell lines
☒	Palaeontology and archaeology
☒	Animals and other organisms
	☒ Clinical data
☒	Dual use research of concern
☒	Plants

Methods

n/a	Involved in the study
☒	ChIP-seq
	☒ Flow cytometry
☒	MRI-based neuroimaging

Antibodies

Antibodies used

The following antibodies were used for the Activation Induced Marker (AIM) Assay: anti-CD4-FITC (BD Biosciences, cat 345768, clone SK3, lot 3237669); dilution 1:100, anti-OX40-PE (BioLegend, cat 350004, clone ACT35, lot B367290); dilution 1:20, anti-CD69-PE-Cy7 (BioLegend, cat 310912, clone FN50, lot B389737); dilution 1:20, anti-CD137-APC Fire 750 (BioLegend, cat 309834, clone 4B4-1, lot B372488); dilution 1:20, anti-CD8-PB (BioLegend, cat 301023, clone RPA-T8, lot B413305); dilution 1:50, anti-CD3-BV510 (BioLegend, cat 300447, clone UCHT1, lot B358291); dilution 1:200.
For the proliferation assay, the following antibodies were used: anti-CD4-PerCP-Cy5.5 (BD, cat 332772, clone SK3, lot 3342745); dilution 1:25, anti-OX40-PE (BioLegend, cat 350004, clone ACT35, lot B367290); dilution 1:20, anti-CD69-PE-Cy7 (BioLegend, cat 310912, clone FN50, lot B389737); dilution 1:20, anti-CD137-APC Fire 750 (BioLegend, cat 309834, clone 4B4-1, lot B372488); dilution 1:20, anti-CD8-APC (BioLegend, cat 344722, clone SK1, lot B401698); dilution 1:50, anti-CD3-FITC (E-bioscience, cat 11-0036-42, clone SK7, lot 2452783); dilution 1:25.

Validation

The manufacturer's specifications for each antibody was reviewed to ensure these antibodies were suitable for our application.
Each primary antibody is supported as follows: the manufacturer provides flow-cytometry (or IVD) validation data and/or the clone is broadly cited and referenced in ≥20–100 peer-reviewed publications. Some are confirmed to stain activated vs. resting populations (e.g. BioLegend FN50 on CD69); clones such as SK3, UCHT1, SK1/RPA-T8 and ACT35 are gold standards across immunophenotyping studies.

Specifically:
-Anti-CD4-FITC (BD Biosciences 345768, clone SK3)
Manufacturer's validation (BD): IVD-validated for human peripheral blood/T cells, specific to CD4; commonly used on BD FACS systems (https://www.bdbiosciences.com/en-eu/products/reagents/flow-cytometry-reagents/clinical-diagnostics/single-color-antibodies-asr-ivd-ce-ivd/cd4-fitc.345768?tab=product_details)
Literature: cited in >100 publications (https://www.citeab.com/antibodies/2414697-345768-bd-cd4-fitc?); workflows describe reliable gating of CD3+CD4+ lymphocytes.
-Anti-OX40-PE (BioLegend 350004, clone ACT35)
Manufacturer: PE conjugate of clone ACT35; BioLegend confirms flow cytometry validation and quality testing (https://www.biocompare.com/9776-Antibodies/2237185-PECy7-antihuman-CD134-OX40/?)
Literature: applied in studies investigating OX40 expression on activated T cells and immune phenotyping (https://www.nature.com/articles/s41416-020-0810-1?)
-Anti-CD69-PE-Cy7 (BioLegend 310912, clone FN50)
Manufacturer: PE-Cy7 conjugate, targets 27–33-kDa early-activation antigen; flow cytometry validated (https://www.biolegend.com/nl-nl/products/pe-cyanine7-anti-human-cd69-antibody-1918?)
Literature: widely used as activation marker in both basic and translational research (https://www.biocompare.com/pfu/110447/soids/1219/Antibodies/CD69?)
-Anti-CD137-APC-Fire 750 (BioLegend 309834, clone 4B4-1)
Manufacturer: BioLegend's APC Fire 750 variants are flow-validated.
Literature: Clone 4B4-1 widely used to detect CD137 (4-1BB) on activated T cells in numerous FC studies (reproduction in literature is standard) (https://www.biolegend.com/de-at/products/apc-fire-750-anti-human-cd137-4-1bb-antibody-16023?)
-Anti-CD8-PB (BioLegend 301023, clone RPA-T8)
Manufacturer: PB-labelled RPA-T8 clone for human CD8; typical flow-cytometry reagent from BioLegend with expected spec.
Literature: RPA-T8 clone is one of the most widely used for CD8 T cell phenotyping across immunology research (https://www.biocompare.com/Product-Reviews/186762-A-very-good-CD8-antibody-for-flow-cytometry-from-BioLegend-PE-conjugated-monoclonal-mouse-anti-human-CD8-alpha-clone-RPA-T8/?)
-Anti-CD3-BV510 (BioLegend 300447, clone UCHT1)
Manufacturer: BV510-labeled clone UCHT1; widely marketed and validated for flow cytometry (https://www.biolegend.com/en-ie/products/brilliant-violet-510-anti-human-cd3-antibody-9792?)
Literature: UCHT1 is the classic CD3 clone for T-cell gating in FC panels; used extensively in the literature.
-Anti-CD4-PerCP-Cy5.5 (BD 332772, clone SK3)
Manufacturer: Analogous to FITC version, but PerCP-Cy5.5 conjugate; IVD/FC validated by BD.
Literature: SK3 clone widely cited and consistent performance across fluorochromes. This reagent has been cited in 37–78 peer-reviewed publications, underscoring its widespread use and reliability (https://www.citeab.com/antibodies/2414593-332772-bd-cd4-percp-cy-5-5?)
-Anti-CD8-APC (BioLegend 344722, clone SK1)
Manufacturer: APC-labeled SK1 clone targeting human CD8; FC-validated reagent.
Literature: SK1 is standard clone for CD8, referenced in many FC panels (https://www.biocompare.com/9776-Antibodies/1077914-

APC-antihuman-CD8/?)
- Anti-CD3-FITC (eBioscience/Thermo Fisher 11-0036-42, clone SK7)
Manufacturer: FITC-SK7 clone, pre-titrated for flow cytometry (https://www.thermofisher.com/antibody/product/CD3-Antibody-clone-SK7-Monoclonal/11-0036-42?)
Literature: SK7 clone commonly used for CD3 gating; standard in immunophenotyping protocols.
Moreover, we validated our results using a positive (SEB) and negative (medium DMSO) control. Unfortunately, due to a limited amount of available PBMCs we were unable to include a positive control for every sample (time point), but we ensured a positive control was used for every participant. A negative control was used for every sample.

Clinical data

Policy information about clinical studies
All manuscripts should comply with the ICMJE guidelines for publication of clinical research and a completed CONSORT checklist must be included with all submissions.

Clinical trial registration: The present study on itself is not a clinical trial, however we used stored samples from the Primo-SHM trial. The parent study (Primo-SHM trial) was registered with the number ISRCTN59497461.

Study protocol: The protocol of the parent study (Primo-SHM trial) and the CONSORT checklist can be accessed as a supplement to the published paper that describes the results of the trial (PMID: 22479156).

Data collection: The parent study (Primo-SHM trial) was a multicenter, open-label randomized controlled trial comparing temporary early cART (24 or 60 wk) with no treatment. Patients were recruited in 13 HIV treatment centers in the Netherlands. Participants were recruited from May 2003 until March 2010, and follow-up data were collected until April 2015.

Outcomes: The primary efficacy end points of the parent study (Primo-SHM trial) were (1) the viral set point, defined as pVL at 36 wk after randomization in the no treatment arm and pVL at 36 wk after TI in the treatment arms, and (2) the total time that patients were off therapy, defined as the time between randomization and start of cART in the no treatment arm, and as the time between TI and restart of cART in the treatment arms. The results of the trial are published previously (PMID: 22479156).

Plants

Seed stocks: *Report on the source of all seed stocks or other plant material used. If applicable, state the seed stock centre and catalogue number. If plant specimens were collected from the field, describe the collection location, date and sampling procedures.*

Novel plant genotypes: *Describe the methods by which all novel plant genotypes were produced. This includes those generated by transgenic approaches, gene editing, chemical/radiation-based mutagenesis and hybridization. For transgenic lines, describe the transformation method, the number of independent lines analyzed and the generation upon which experiments were performed. For gene-edited lines, describe the editor used, the endogenous sequence targeted for editing, the targeting guide RNA sequence (if applicable) and how the editor was applied.*

Authentication: *Describe any authentication procedures for each seed stock used or novel genotype generated. Describe any experiments used to assess the effect of a mutation and, where applicable, how potential secondary effects (e.g. second site T-DNA insertions, mosiacism, off-target gene editing) were examined.*

Flow Cytometry

Plots

Confirm that:

- [x] The axis labels state the marker and fluorochrome used (e.g. CD4-FITC).
- [x] The axis scales are clearly visible. Include numbers along axes only for bottom left plot of group (a 'group' is an analysis of identical markers).
- [x] All plots are contour plots with outliers or pseudocolor plots.
- [x] A numerical value for number of cells or percentage (with statistics) is provided.

Methodology

Sample preparation: Peripheral blood was collected by venous puncture into Vacutainer Tubes with Sodium Heparin. Peripheral blood mononuclear cells (PBMCs) were isolated from Vacutainer Tubes with Sodium Heparin using Ficoll-Isopaque density gradient centrifugation and cryopreserved vapor phase of liquid nitrogen for future analysis. For the Activation Induced Marker (AIM) assay and proliferation assay, cryopreserved PBMCs were thawed and stimulated with HIV peptide pools (described in Methods section).

Instrument: Fluorescence was measured on the FACS Canto II fluorescence-activated cell sorter (BD Biosciences).

Software: Marker expression levels were analyzed using FlowJo version 10.8.1 (TreeStar, Ashland, OR, USA).

Cell population abundance: n/a

Gating strategy

The general gating strategy used in the analyses was as follows:
Activation Induced Marker (AIM) Assay:
morphology (FSC-A/SSC-A) > singlets (FSC-A/FSC-H) > CD3+ population (SSC-A/CD3- BV510) > CD4/CD8 (CD4-FITC / CD8-PacificBlue)>> reactive CD4+ T cells (CD137 APC Fire750 / OX40 PE) and reactive CD8+ T cells (CD137 APC Fire750 / CD69 PE-Cy7).
Proliferation assay:
morphology (FSC-A/SSC-A) > singlets (FSC-A/FSC-H)) > CD3+ population (SSC-A/CD3- BV510) > CD4/CD8 (CD4-FITC / CD8-PacificBlue >> proliferating CD4+ T cells (CellTrace Violet) and proliferating CD8+ T cells (CellTrace Violet).
The proportion of proliferating cells was determined using FlowJo V10 (FlowJo). The precursor frequency was calculated as follows: per generation the amount of CD8+ T-cells that proliferated were calculated (number of cells * 2(generation)); the precursor frequency is the total number of CD8+ T-cells that proliferated per 100 CD8+ T-cells (total CD8+ T cells).

☒ Tick this box to confirm that a figure exemplifying the gating strategy is provided in the Supplementary Information.
